# Research progress on the interaction mechanisms and functions between exosomes and the cytoskeleton

**DOI:** 10.3389/fcell.2026.1718115

**Published:** 2026-05-04

**Authors:** Shili Yang, Xinyan Zhang, Bo Chen, Haiyang Kou, Lingyan Lai, Huaiquan Liu, Yunling Xu, Yu Sun

**Affiliations:** Guizhou University of Traditional Chinese Medicine, Guiyang, Guizhou, China

**Keywords:** cytoskeleton, exosomes, interaction, mechanism of action, research progress

## Abstract

Exosomes, as key mediators of intercellular communication, play a central regulatory role in cellular physiological and pathological processes through their dynamic interaction with the cytoskeleton. The cytoskeleton is a dynamic network composed of microtubules, microfilaments, and intermediate filaments. Microtubules provide track support for the directional transport of MVBs. Microfilament rearrangement generates contractile forces that promote MVB fusion with the plasma membrane. Therefore, the cytoskeleton directly participates in the biogenesis, intracellular transport, and secretion of exosomes. Moreover, cytoskeletal dynamics, coordinated by molecules such as Rab GTPases, affect exosome secretion efficiency. Conversely, exosomes carry bioactive molecules such as proteins, nucleic acids, and lipids. These molecules can regulate cytoskeletal rearrangement in recipient cells by modulating signaling pathways like the TGF-β/Smad signaling pathway and the RhoA/ROCK signaling pathway. Consequently, they influence target cell functions like morphology maintenance, migration, and proliferation. Dysregulation of this interaction is closely related to the progression of various diseases, including tumors and neurodegenerative diseases. For instance, disrupting the dynamic structure of the cytoskeleton or blocking the cytoskeletal remodeling process can significantly reduce exosome secretion, while abnormal exosome transfer disrupts cytoskeletal homeostasis. Current research still faces challenges, such as unresolved details of the molecular regulatory network and a lack of in-depth mechanistic validation in in vivo models. Future studies need to explore in depth novel regulatory factors and signaling pathways and investigate disease diagnosis and treatment strategies based on this interaction. This will provide a theoretical basis and innovative ideas for the prevention and treatment of related diseases.

## 1 Introduction

**FIGURE 1:**
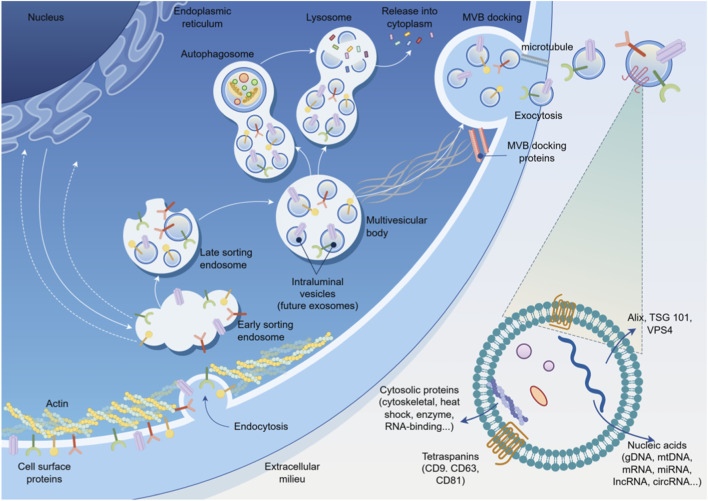
Schematic diagram of the mechanism by which the interaction between exosomes and the cytoskeleton regulates exosome biogenesis and function. Note: This figure shows the whole process of exosomes from biogenesis to intracellular transport, secretion and release, and then to action on recipient cells, as well as the key regulatory role of core components of the cytoskeleton in this process. The core structures of exosome biogenesis are marked in the figure: Early-Sorting Endosome (ESE), Late-Sorting Endosome (LSE), MVB, ILVs; key molecules related to exosome generation: ESCRT complex-related proteins (Alix, TSG101, VPS4), tetraspanins (CD9, CD63, CD81); core functional components of the cytoskeleton: microtubules that provide track support for the directional transport of MVBs, microfilaments that regulate the fusion of MVBs with the plasma membrane; meanwhile, the core contents carried by exosomes (proteins, nucleic acids, etc.), the exocytosis and release process of MVBs, and the pathway of exosomes regulating the function of recipient cells after entering them are also shown. This figure was drawn by Figdraw.

Research on exosomes, the core mediators of intercellular information transfer, began in the 1980s. Initially, their release was considered a pathway for cells to excrete metabolic waste. With deeper scientific exploration, exosomes are now clearly defined as a subclass of extracellular vesicles (EVs), which are cup-shaped or spherical membranous vesicles ranging from 40 to 100 nm in diameter, released into the extracellular space after the fusion of intracellular multivesicular bodies (MVBs) with the plasma membrane. Their hallmark characteristics are the carrying of tetraspanins such as CD9, CD63, CD81, and endosomal sorting-related proteins such as TSG101 and Alix (Pan and Johnstone 1983; Simons and Raposo, 2009; van Niel et al., 2018) which are also the core molecular markers distinguishing exosomes from other EV subtypes. In addition to exosomes, the EV family includes two other major subtypes: microvesicles, which are formed by direct budding and shedding of the plasma membrane without an endosome-derived biogenesis process (Théry et al., 2018; Yáñez-Mó, M. et al., 2015); and apoptotic bodies, which are vesicles produced by cell membrane disintegration during apoptosis and contain intact cellular components such as nuclear fragments and organelles (Battistelli and Falcieri, 2020; Samir et al., 2013). Exosomes can specifically load bioactive molecules such as proteins, nucleic acids, and lipids (Pan and Johnstone 1983; Simons and Raposo 2009). With this key loading property, they play a key role in physiological activities including intercellular signal transduction, the body’s immune response, cellular homeostasis maintenance, and autophagy regulation (Gurunathan et al., 2021). The cytoskeleton is a dynamic three-dimensional network structure composed of microtubules, microfilaments, and intermediate filaments (Fletcher and Mullins 2010). It not only maintains cell shape and mechanical stability but also directly participates in the generation, intracellular transport, and release of exosomes. This participation occurs through kinesin/dynein-mediated vesicle transport processes (Schuh, 2011) and the regulation of exocytosis by actomyosin contraction (Biton et al., 2023). Studies indicate that exosome biogenesis and secretion processes highly depend on precise cytoskeletal regulation: the microtubule network provides track support for the intracellular directional transport of MVBs encapsulating exosome precursors, namely Intraluminal Vesicles (ILVs). Microfilament rearrangement generates contractile forces that promote MVB anchoring and fusion with the plasma membrane (van Niel et al., 2018; Ostrowski et al., 2010). This regulatory process relies on the coordinating role of molecules like Ras-related proteins in brain guanosine triphosphatases (Rab GTPases) in MVB transport, thereby affecting exosome secretion efficiency (Xu et al., 2023). This suggests that cytoskeletal dynamics are a key rate-limiting step in exosome biosynthesis and release. Furthermore, the interaction between exosomes and the cytoskeleton permeates processes like material transport, signal transduction, and cell morphology regulation: exosomes enter target cells via phagocytosis or receptor-mediated endocytosis. This internalization requires the synergistic action of the actin cytoskeleton and Phosphatidylinositide 3-kinases (PI3K) (Feng et al., 2010). After exosomes enter the recipient cell, their encapsulated cargoes, such as microRNAs (miRNAs), are directionally transported along microtubules and microfilament to interact with organelles including the nucleus and endoplasmic reticulum. This interaction regulates gene expression and protein synthesis (Ishikawa et al., 2024). In summary, the interaction between exosomes and the cytoskeleton is not only a core mechanism of intercellular communication but also a key link in maintaining cellular physiological homeostasis and regulating disease progression. Therefore, in-depth investigation into the molecular mechanisms of their interaction holds significant scientific importance. The regulatory relationship between the cytoskeleton and the whole process of exosome biogenesis, intracellular transport, secretion and release, as well as the interaction with recipient cells, is shown in Figure 1.

## 2 Composition, secretion, and biogenesis of exosomes

### 2.1 Composition of exosomes

The molecular composition of exosomes is extremely complex with significant heterogeneity, mainly comprising three types of bioactive molecules: proteins, nucleic acids, and lipids. There is currently no unified standard for the classification of exosomal proteins, which can be roughly divided into common component proteins and specific component proteins according to their expression and distribution characteristics (Zhang et al., 2020). Common component proteins are ubiquitously present in exosomes derived from most cell types, mainly covering the following four subclasses, with the composition and core functions of each subclass as follows: First, membrane fusion and transport-related proteins, mainly including Rab GTPases family proteins, annexins, and heat shock proteins (e.g., HSP60, HSP70, HSP90). The core function of these proteins is to regulate membrane fusion, intracellular trafficking, and exocytosis of vesicles. Second, MVB formation-related proteins (ESCRT complex-related proteins), mainly including the core components of the Endosomal Sorting Complex Required for Transport (ESCRT) such as ALIX, TSG101, and VPS4, which are key molecules regulating the biogenesis of exosome precursors (ILVs). Third, tetraspanins: mainly including CD9, CD63, and CD81, which are hallmark proteins of exosomes. They can participate in the biogenesis of exosomes independent of ESCRT by regulating endosomal membrane curvature and cargo sorting. The first two types of proteins dominate exosome biogenesis, the third type participates in exosome formation and sorting, and the above three types of proteins jointly participate in the whole process regulation of exosome formation, intracellular transport and release (Skotland et al., 2019; Li et al., 2023). Fourth, structure and function-related proteins, mainly including integrins, actin, myosin, cofilin, tubulin, etc., which not only participate in maintaining the basic structure of exosomes, but also are key functional molecules for exosomes to regulate cytoskeletal rearrangement in recipient cell (Zhang et al., 2020; Dai et al., 2020). Specific component proteins depend on the type and conditions of the exosome-secreting cell (Feng et al., 2010). For instance, cyclic guanosine monophosphate (cGMP)-dependent protein kinase G1 (PKG1) and Nuclear transcription factor X-box binding protein 1 (NFX1) are detected only in plasma exosomes (Chen et al., 2017), while Epidermal Growth Factor Receptor (EGFR) is significantly highly expressed on the surface of exosomes secreted by lung cancer cells (Jakobsen et al., 2015). These molecules confer parental cell functional specificity to exosomes and can also serve as biomarkers for disease diagnosis.

The nucleic acid composition of exosomes exhibits complex diversity, containing various types of Ribonucleic Acid (RNA) and DeoxyriboNucleic Acid (DNA). Its specific composition varies depending on the cell source, physiological state, and pathological environment. The most abundant RNA types in exosomes include Micro Ribonucleic Acid (miRNA), Messenger RNA (mRNA), Long non-coding RNA (lncRNA), and Circular Ribonucleic Acid (CircRNA) (Yu et al., 2022; Gurunathan et al., 2019; Lin H et al., 2021). Among them, miRNA is a type of non-coding RNA approximately 22 nucleotides in length (Bartel, 2004), which is one of the most characteristic nucleic acid components in exosomes (Valadi et al., 2007). After exosomes deliver miRNAs to recipient cells, mature miRNAs can specifically bind to the 3′untranslated region (3′UTR) of target mRNAs through base complementary pairing (Zomer et al., 2010), and then regulate post-transcriptional gene expression through two core ways: one is to directly induce the degradation of target mRNAs, and the other is to inhibit the translation process of target mRNAs, ultimately silencing the expression of target genes at the post-transcriptional level (Bartel, 2004; Brennecke et al., 2005). For example, in the tumor microenvironment, exosomal miRNA can promote tumor progression by inhibiting tumor suppressor genes or activating oncogenes (Xie et al., 2022). mRNA can be transported by exosomes to recipient cells and translated into proteins, participating in intercellular functional regulation. For instance, exosomal mRNA from tumor cells can convey coding information for drug resistance-related proteins (Lin et al., 2015). lncRNA is a non-coding RNA longer than 200 nucleotides. It regulates gene expression by interacting with DNA, RNA, or proteins. Its abnormal expression in exosomes is associated with the occurrence and development of various diseases. For example, the expression levels of specific lncRNAs change significantly in serum exosomes of endometriosis patients (Huang et al., 2024). circRNA is a type of non-coding RNA with a covalently closed circular structure. It exhibits high stability and tissue specificity. Exosomal circRNA can participate in crosstalk between tumors and between tumors and stroma in the tumor microenvironment and holds promise as a biomarker for cancer diagnosis, prognosis, and prediction (Lin H et al., 2021). Besides the above RNAs, exosomes also contain other non-coding RNAs, such as Piwi-interacting RNA (piRNA), Small Nuclear RNA (snRNA), Small Nucleolar RNA (snoRNA), and Transfer Ribonucleic Acid (tRNA) fragments (Pantazi et al., 2022). DNA in exosomes mainly includes Genomic DNA (gDNA) and human mitochondrial DNA (mtDNA) (Lin H et al., 2021). gDNA usually exists in fragmented form, containing genetic information from the cell source. For example, exosomal gDNA from tumor cells can carry specific gene mutations, serving as a liquid biopsy marker (Yu, D. et al., 2022). The cGAS-STING pathway is the core sensing pathway for cytoplasmic DNA. Not only can exosome-carried mtDNA activate this pathway, but double-stranded DNA (dsDNA) within exosomes can also be recognized by cGAS, thereby activating the STING-dependent innate immune cascade, which plays a key role in tumor immune regulation, antiviral response and other processes (Lin H et al., 2021; Kalluri 2016; Yu and Liu, 2021). It is noteworthy that the nucleic acid composition of exosomes is dynamically changing. Its abundance and function can change significantly with cellular physiological state or pathological stimuli. For instance, M1 macrophage exosomes have a relatively high abundance of miRNA, while M2 macrophage exosomes are rich in tRNA fragments. This difference may be related to their distinct roles in inflammation regulation (Pantazi et al., 2022).

The lipid components of exosomes mainly include phosphatidylserine (PS), sphingomyelin (SM), cholesterol (CHOL), glycosphingolipids, phosphatidylethanolamine (PE), phosphatidylcholine (PC), and phosphatidylinositol (PI). PS on the exosome membrane is often exposed on the surface, and its content is significantly higher than that in the plasma membrane of parental cells. For example, abnormal PS exposure in exosomes derived from ovarian cancer cells can serve as a biomarker for cancer diagnosis (Lea, J. et al., 2017). SM and CHOL are core components of the exosome membrane. Studies show that the content of SM and CHOL in exosomes is 2–3 times higher than in parental cells, forming lipid raft-like structures. These not only maintain membrane stability but also participate in exosome biogenesis and intercellular communication (Kalluri and LeBleu, 2020; Guo and Zhang 2022). For instance, cholesterol promotes the fusion of exosomes with target cell membranes by regulating membrane fluidity and rigidity, enabling efficient cargo delivery (Zhuo et al., 2024; Palmulli et al., 2024). PE and PC, as major phospholipid components, participate in the structural constitution of the exosome membrane. The externalization of PE can trigger exosome budding and release (Mathieu et al., 2019), while PC regulates the targeted delivery efficiency of exosomes by interacting with specific ligands (Wang et al., 2025). PI and its derivatives, such as phosphoinositides, play a key role in regulating exosome release and membrane dynamics. For example, they influence exosome secretion efficiency by regulating secretory autophagy pathways (Skotland et al., 2019). Furthermore, interactions exist between exosome components. For instance, tetraspanins like CD63 sort cholesterol into intraluminal vesicles of endosomes, forming transient storage pools. This regulates intracellular cholesterol distribution and also delivers cholesterol to recipient cells via exosomes, affecting their metabolic state (Palmulli et al., 2024). The three core components of exosomes—proteins, nucleic acids, and lipids—not only determine its structural stability and the functional specificity of intercellular signal transmission, but also serve as the molecular basis for its interaction with the cytoskeleton. Among them, cytoskeleton-related proteins such as actin and tubulin, as well as lipid components that regulate membrane dynamics, are the material basis for the interaction between exosomes and the cytoskeleton. The heterogeneity and dynamic change characteristics of exosome composition also provide a molecular premise for the cell/environment-specific regulation of the interaction between the two.

### 2.2 Biogenesis process of exosomes

Exosome biogenesis begins with plasma membrane deformation and endocytosis: the asymmetric distribution of clathrin, caveolin, lipid multimeric proteins, and proteins containing the Bin-Amphiphysin-Rvs (BAR) domain can synergistically drive the formation and stabilization of the initial membrane curvature of the plasma membrane (Hubert et al., 2020; Sharma et al., 2004). Among them, BAR domain proteins can sense and induce membrane bending through their banana-shaped dimer structure, and are the core regulatory molecules for plasma membrane deformation and Early-Sorting Endosome (ESE) generation during endocytosis (Peter et al., 2004; Carman and Dominguez, R., 2018; Worby and Dixon, 2002); ESEs further develop into Late-Sorting Endosomes (LSEs) after maturation (Huotari and Helenius, 2011). The LSE membrane invaginates to enwrap substances like proteins, lipids, and nucleic acids, forming ILVs. This late endosome structure enriched with ILVs is defined as the MVB (Henne et al., 2013; Colombo et al., 2014). MVB formation primarily occurs through two mechanisms: the ESCRT-dependent pathway and the ESCRT-independent pathway. In the ESCRT-dependent pathway, this process is executed cooperatively by four subcomplexes (ESCRT-0, -I, -II, -III) and accessory proteins like Vacuolar Protein Sorting 4 (VPS4) and ALIX: ESCRT-0 (containing HRS and STAM1/2) recognizes and recruits ubiquitinated membrane protein cargo to the endosomal membrane via its ubiquitin-binding domain, while also recruiting ESCRT-I (e.g., TSG101, VPS28, etc.). ESCRT-I further recruits ESCRT-II (e.g., VPS22, VPS25, VPS36). Together, they induce endosomal membrane deformation and bud formation. Subsequently, ESCRT-III (e.g., VPS20, SNF7, VPS2, VPS24) assembles into a spiral-shaped polymer at the membrane neck, driving membrane scission to form ILVs. Finally, the VPS4 ATPase hydrolyzes ATP to disassemble the ESCRT-III complex, enabling its recycling (Ostrowski et al., 2010; Henne et al., 2011; Baietti et al., 2012; Colombo et al., 2013). In the ESCRT-independent pathway, neutral sphingomyelinase 2 (N-SMase2) hydrolyzes sphingomyelin to produce ceramide. The conical structure of ceramide drives spontaneous membrane bending, leading to ILV formation (Choezom and Gross, 2022). Additionally, tetraspanins like CD63, CD9, and CD81 regulate the loading of specific cargo by organizing lipid microdomains, thereby promoting membrane curvature changes and ILV formation (van Niel et al., 2011; Hessvik and Llorente, 2018). For example, CD63 directly facilitates the entry of premelanosome protein (PMEL) into ILVs, while CD9 enhances the exosomal delivery efficiency of metalloproteinases by binding to them (Sung et al., 2020). Furthermore, ESCRT-associated proteins like ALIX can participate directly in ILVs biogenesis independently of the classical ESCRT complex, through interactions with Syndecan Binding Protein (SDCBP) and tetraspanins (Colombo et al., 2014; Colombo et al., 2013). The formed MVBs have two fates: most fuse with lysosomes, leading to cargo degradation; a minority are transported near the plasma membrane under the regulation of Rab GTPases (e.g., Rab27a/b). These MVBs then release ILVs as exosomes into the extracellular environment through a membrane fusion process mediated by the Soluble N-ethylmaleimide-sensitive factor Attachment protein REceptor (SNARE) complex protein Vesicle-Associated Membrane Protein 7 (VAMP7) (Ostrowski et al., 2010; Kalluri and LeBleu, 2020; Hessvik and Llorente, A., 2018; Fader et al., 2009). Exosome biogenesis is a multi-step and highly ordered membrane remodeling process. From early endosome formation to MVB maturation and ILVs generation, ESCRT-dependent and independent pathways jointly complete the biosynthesis and cargo loading of exosomes. In this process, the maturation of endosomes and the intracellular spatial localization of MVBs are highly dependent on the support and regulation of the cytoskeleton system, which lays a biological process foundation for the subsequent elaboration of the interaction mechanism between the two.

### 2.3 Regulatory mechanism of exosome secretion and release

Exosome secretion is a highly ordered biological process that is precisely regulated in multiple dimensions. The whole process starts with the fate decision of mature MVBs, followed by sequential steps of intracellular directional transport, plasma membrane docking and fusion, and finally completes vesicle release. The rigorous regulation of this process directly determines the secretion level of exosomes and their biological functions (Xu et al., 2023). Specifically, mature MVBs mainly have two distinct cellular fates, and the choice of these two fates is directly related to the generation and release of exosomes: one is to fuse with lysosomes, and the contents they carry are degraded by lysosomal hydrolases, thereby participating in cellular material circulation; the other is to be directionally transported to the plasma membrane, and the ILVs contained therein are released through exocytosis, and these ILVs released into the extracellular space are exosomes (van Niel et al., 2018; Kowal et al., 2016). Among them, ceramide can specifically induce the budding of ILVs into the MVB cavity, providing a key structural basis for the initial generation of exosomes (Trajkovic et al., 2008). It is worth noting that the ratio of the two fates of MVBs is not fixed, but has a high degree of cell type specificity: in most non-specialized cells, most MVBs are directed to the lysosomal pathway for degradation, and the proportion of MVBs with exosome secretion function is relatively low; while in special cell types such as immune cells, stem cells or tumor cells, the proportion of MVBs that can secrete exosomes will be significantly increased (Colombo et al., 2014; Baietti et al., 2012). The fate trend of MVBs is not random, but is synergistically regulated by a variety of molecular mechanisms, including Rab GTPases, SNARE protein, ESCRT complex and membrane contact sites (Colombo, M. et al., 2014; Baietti et al., 2012; Verweij et al., 2022; Krylova and Feng 2023). Specifically, the ESCRT complex-related pathway is directly involved in exosome biogenesis and MVB fate regulation (Colombo et al., 2014; Baietti et al., 2012); ER membrane contact sites can indirectly regulate MVB fate decision and exosome secretion process by supporting the conversion of endosomal small GTPases (Verweij et al., 2022). These regulatory factors jointly determine the final direction of MVBs - whether they are directed to lysosomes for degradation, or transported to the plasma membrane to secrete exosomes. Among them, the regulatory method of inhibiting endolysosome fusion can directly improve the secretion efficiency of exosomes (Shelke et al., 2023). Among many regulatory molecules, Rab GTPases family proteins are the core molecular switches that regulate MVB transport, anchoring and fusion. Among them, Rab7 is the key regulatory factor that mediates the lysosomal degradation pathway of MVBs. Activated (GTP-bound) Rab7 can interact with effector protein RILP and cholesterol-sensitive protein ORP1L, so that the p150Glued subunit of dynactin forms a complex with it, and then recruits the dynein complex to mediate the transport of MVBs to the minus end of microtubules (perinuclear lysosome enrichment area); finally, with the synergistic assistance of Homotypic Fusion and Vacuole Protein Sorting (HOPS) complex and RILP, it promotes the fusion of MVBs with lysosomes and completes degradation (Xu et al., 2023). In addition to Rab7, the conversion process of phosphatidylinositol is also involved in the regulation of MVB fate: when the phosphatidylinositol conversion occurs on the MVB membrane, it is not the direct conversion of PI(3)P to PI(4)P, but myotubularin 1 (MTM1, a PI(3)P phosphatase) first hydrolyzes PI(3)P to phosphatidylinositol (PI), and then phosphatidylinositol four kinase IIα (PI4KIIα) phosphorylates PI to PI(4)P. This sequentially catalyzed conversion process recruits the exocyst complex, thereby inhibiting the lysosomal targeting of MVBs and driving their secretory transport to the plasma membrane (Liu et al., 2023). In addition, the BLOC-1 Related Complex (BORC)-ARL8-HOPS axis is also the core pathway that regulates the fusion of MVBs with lysosomes: the BORC complex is responsible for recruiting the small GTPase ARL8 to the lysosomal membrane, and ARL8 further recruits the HOPS complex to promote the tethering and fusion of MVBs with lysosomes; inactivation of this pathway can significantly inhibit the degradation pathway of MVBs, allowing more MVBs to enter the secretory pathway, thereby up-regulating the secretion level of exosomes (Shelk et al., 2023). When MVBs are determined to go to the secretory pathway, the directional transport of secretory MVBs to the plasma membrane is highly dependent on the synergistic effect of the cytoskeleton system and molecular motor proteins. Microtubules, as the core intracellular transport track, their polar structure (the plus end faces the cell edge, and the minus end faces the microtubule organizing center) provides a solid physical basis for the long-distance directional transport of MVBs. During the transport process, Rab GTPases on the MVB membrane need to use adaptor proteins as intermediaries to recruit and bind kinesin family proteins, thereby mediating the plus-end transport of MVBs along microtubules to the cell periphery; while dynein is responsible for mediating the reverse perinuclear transport of MVBs. The dynamic balance between kinesin and dynein directly determines the intracellular spatial distribution and transport efficiency of MVBs (Xu et al., 2023). Furthermore, the Rab GTPases family plays a core spatiotemporal regulatory role in the process of MVB transport and exosome secretion, and precisely regulates the whole process spatiotemporal dynamics of MVBs from transport to fusion with the plasma membrane through the cyclic GTP/GDP binding state (Ostrowski et al., 2010; Hsu et al., 2010). Among them, Rab27a and Rab27b are classic molecules that regulate exosome secretion, with a clear division of labor, controlling different steps of the exosome secretion pathway (Ostrowski et al., 2010): Rab27a is mainly responsible for the docking process of MVBs with the plasma membrane, while Rab27b does not participate in docking, but mainly mediates the transport of MVBs from the perinuclear region to the cell periphery. Silencing Rab27b will lead to the redistribution of multivesicular endosomes (MVE) to the perinuclear region and reduce the density of MVE under the cell membrane (Ostrowski et al., 2010). Both Rab27a and Rab27b need to bind to the corresponding effector proteins to exert their regulatory effects, among which Slac2b (Exophilin 5), as the specific effector protein of Rab27b, can participate in the exocytosis process of MVE in HeLa cells (Ostrowski et al., 2010; Fukuda, 2013). Existing studies have confirmed that the functional loss of both Rab27a and Rab27b can significantly inhibit exosome secretion, and this conclusion has been verified in a variety of cell lines (Ostrowski, M. et al., 2010; Bobrie et al., 2011). In addition to the Rab27 subfamily, Rab35, Rab11a and other Rab GTPases family members also participate in the regulation of the classical exosome secretion pathway: among them, Rab35 regulates the recruitment and docking of endosomal vesicles to the plasma membrane through its GTPase activating protein TBC1D10A-C, thereby regulating exosome secretion (Hsu, C. et al., 2010); Rab11a participates in the transport and release of MVBs derived from recycling endosomes, and the exocyst complex can regulate exosome biogenesis through Rab11a (Bai et al., 2022). At the same time, the exocyst complex itself also plays an important role in the process of polarized exocytosis, providing the necessary molecular basis for exosome secretion (He and Guo, 2009). Therefore, the biogenesis and secretion of exosomes are a multi-step, highly ordered dynamic process early endosome formation, MVB maturation and fate decision, to intracellular directional transport of MVBs, plasma membrane anchoring and fusion release, every key link is closely related to the dynamic regulation of the cytoskeleton system (Xu et al., 2023; Yeat and Chen, 2025; Robinson et al., 2024; Mittelbrunn et al., 2015). The cytoskeleton not only provides structural support and power basis for the core steps such as intracellular transport and membrane fusion of exosomes, but also has a complex two-way interaction with them, and their dynamic balance is the key to regulating cell physiological functions and disease occurrence and development.

## 3 Interaction between cytoskeleton and exosomes

### 3.1 Structure and function of cytoskeleton

The cytoskeleton is a three-dimensional network composed of microtubules, microfilaments, and intermediate filaments. It plays a central role in maintaining cell shape, material transport, cell movement, and signal transduction (Théry et al., 2018). Microtubules are polymerized from α- and β-tubulin heterodimers, forming hollow tubular structures approximately 25 nm in diameter. These heterodimers are arranged head-to-tail to form protofilaments, typically with 13 protofilaments laterally connected to form the microtubule wall (Manka and Moores, 2018; Goodson and Jonasson, 2018). The tubular polarity is determined by the directionality of α-tubulin (minus end) and β-tubulin (plus end) (Knossow et al., 2020). Dynamic instability is a core characteristic of microtubules, manifested as the alternating occurrence of rapid polymerization and depolymerization. This process is driven by GTP hydrolysis bound to β-tubulin (Knossow et al., 2020; Kalutskii et al., 2025). Microtubules have diverse physiological functions. During cell division, microtubules assemble into the spindle apparatus. They dynamically capture chromosome kinetochores and mediate their separation, ensuring precise distribution of genetic material (Lim et al., 2024). Moreover, the central microtubule complex, relying on the active regulatory mechanism of kinesin and coordinating with dynein, enables ciliary beating, playing a key role in cell movement and signal transduction (Han et al., 2022). Simultaneously, microtubules serve as tracks for molecular motors like kinesin and dynein, providing support for the long-distance directional transport of organelles such as mitochondria and vesicles. Their transport efficiency is regulated by microtubule post-translational modifications (e.g., acetylation, polyglutamylation) and the coordinated action of motor proteins (Barlan and Gelfand, 2017). Microfilaments, also known as microfilament, are core components of the eukaryotic cytoskeleton. They are formed by the polymerization of globular actin monomers (G-actin) into fibrous polymers (F-actin) approximately 7 nm in diameter. They exhibit high dynamics and polarity (Oosterheert et al., 2022; Chou and Pollard, 2019; Pollard, 2016). The basic structural unit, G-actin, has a molecular weight of approximately 42 kDa. It contains four subdomains and a central cleft that binds ATP/ADP and Mg2+ ions. Its three-dimensional structure is highly conserved evolutionarily (Chou and Pollard 2019; Pollard, 2016). The polarity of microfilaments is determined by the asymmetric arrangement of G-actin monomers, featuring a fast-growing barbed end (plus end) and a slow-growing pointed end (minus end). This polarity directly influences microfilament dynamic assembly and interactions with other proteins (Chou and Pollard, 2019; Pollard, 2016). Functionally, as a core component of the cytoskeleton, microfilaments maintain cell shape and provide mechanical support. They also drive cell movement. For example, migrating neural precursor cells generate thrust through the branched microfilament network mediated by the Actin Related Protein 2/3 Complex (Arp2/3) complex. Coronin family proteins (e.g., *C. elegans* POD-1) regulate microfilament polarity through debranching, ensuring the directionality of cell membrane remodeling (Xie et al., 2021). During cell division, microfilaments and microtubules function synergistically: the formation of the microfilament contractile ring during cytokinesis depends on myosin II activity. Centrosomal protein (Oral-Facial-Digital syndrome 1, OFD1) monitors cell cycle progression by regulating actin branch dynamics. Its absence leads to cytokinesis failure and polyploidy formation (Cao et al., 2023). Furthermore, microfilaments participate in intracellular material transport. For instance, myosin V (MyoV), as a primary motor for transporting cellular components like organelles, vesicles, protein complexes, and mRNA, uses microfilaments as tracks. It achieves directional cargo transport through a self-inhibition release mechanism (Niu et al., 2022). Intermediate filaments (IFs) are a diverse class of cytoskeletal components with significant cell-type specificity. Their unique mechanical properties—including excellent elasticity, tensile strength, and unique stiffening behavior under strain and compression—are crucial for cellular mechanical resilience and tissue integrity (Pogoda et al., 2022; Rölleke et al., 2023). Intermediate filaments have a diameter of about 10 nm, intermediate between microfilaments and microtubules. They are primarily composed of various tissue-specific proteins, including keratins, vimentin, desmin, Glial Fibrillary Acidic Protein (GFAP), neurofilaments, and lamins (Eriksson et al., 2009). All intermediate filament proteins possess a conserved α-helical rod domain flanked by variable N-terminal “head” and C-terminal “tail” domains (Eldirany et al., 2021; Herrmann and Aebi, 2016). Functionally, intermediate filaments maintain cellular homeostasis and mechanical integrity through multiple mechanisms. They link adjacent cells into a whole by anchoring cell junction structures like desmosomes and hemidesmosomes, providing mechanical support to epithelial tissues (Sanghvi-Shah and Weber, 2017). They also participate in nuclear anchoring and organelle positioning (Herrmann et al., 2007) and are involved in intracellular signal transduction. For example, vimentin transmits extracellular mechanical signals to the nucleus by interacting with integrins and cadherins, regulating gene expression (Sanghvi-Shah and Weber, 2017). Additionally, phosphorylation modifications of intermediate filaments dynamically regulate their network structure. For instance, phosphorylation of the vimentin head domain affects its interaction with microtubules, thereby regulating cell migration (Eldirany et al., 2021; Viedma-Poyatos et al., 2020). The three major cytoskeletal components, microtubules, microfilaments, and intermediate filaments, with their unique structural and dynamic characteristics, form a three-dimensional network system with complementary functions and synergistic effects. Among them, the polar track characteristics of microtubules, the dynamic contraction and membrane remodeling ability of microfilaments, and the mechanical support and network stabilization function of intermediate filaments correspond to the core requirements of exosome intracellular transport, membrane fusion and release, and intracellular homeostasis maintenance, respectively. From the structural and functional level, it suggests the innate adaptability of the cytoskeleton to regulate the biological process of exosomes.

### 3.2 Influence of cytoskeleton on exosomes

Microfilaments, microtubules, and intermediate filaments within the cytoskeleton play key roles in the biogenesis, transport, and secretion of exosomes through different mechanisms, particularly determining the transport of MVBs to the plasma membrane (Hoshino et al., 2013; Mittelbrunn et al., 2011). Microfilaments regulate MVB transport and exocytosis through dynamic reorganization. They also influence MVB anchoring and fusion by modulating the density of the subcortical actin layer beneath the plasma membrane. For example, during invadopodia formation, remodeling of the microfilament cytoskeleton promotes directional migration of MVBs to the plasma membrane and exosome release (Hoshino et al., 2013). The actin-binding protein Cortactin promotes branched microfilament formation by activating the Arp2/3 complex, thereby stabilizing MVB docking sites at the plasma membrane and enhancing exosome release (Sinha et al., 2016). Studies show (Yang et al., 2024) that applying magnetic iron oxide nanoparticles to cells can induce cytoskeletal reorganization and microfilament stress fiber formation via magnetic force. This prompts vesicle repositioning, thereby promoting exosome generation to some extent. Furthermore, Protein Kinase C θ (PKCθ) can phosphorylate actin regulatory proteins, reducing microfilament density in MVB aggregation regions. This promotes MVB fusion with the plasma membrane (Calvo and Izquierdo, 2021). Microtubules serve as the primary “track system” within cells. Their polar structure (plus ends oriented towards the cell periphery, minus ends towards the Microtubule Organizing Center) provides the fundamental pathway for directional MVB transport. For instance, kinesin participates in the retrograde transport of early endosomes, ensuring the correct positioning of MVBs within the cell. Dynein interacts with Rab Interacting Lysosomal Protein (RILP) and Rab7 to transport MVBs from the cell periphery towards the microtubule minus end. These two motor systems work synergistically to maintain the dynamic balance of MVBs within the cell (Hoshino et al., 2013; Sinha et al., 2016; Martín-Cófreces et al., 2014; Olenick and Holzbaur, 2019; Soppina, et al., 2022; Villari et al., 2020). The stability and polar arrangement of microtubules directly affect MVB transport efficiency. Their dynamic changes can be achieved by regulating the activity of Microtubule-Associated Proteins (MAPs) (Baas et al., 2006). Research shows (Knudsen et al., 2023) that the microtubule depolymerizing agent nocodazole can inhibit the vesicle transport and exocytosis of Glucose Transporter Type 4 (GLUT4) by disrupting the microtubule network; the vesicle transport of GLUT4 shares a microtubule-dependent intracellular transport system with exosomes, so this process can indirectly inhibit the release of exosomes. Simultaneously, the movement of MVBs along microtubules is also partially regulated by cellular cholesterol content (Record et al., 2014). Vimentin, among intermediate filaments, binds to key proteins in the vesicle membrane transport machinery. This helps provide tracks for vesicle transport, participates in the positioning of endosomes and lysosomes, maintains the integrity of various organelles, and drives exosome-mediated epithelial-mesenchymal transition (Styers et al., 2006; Margiotta and Bucci, 2016; Rahman et al., 2016). Studies indicate (Wu et al., 2021) that vimentin promotes exosome release. The vimentin-binding compound R491 can block exosome release by targeting vimentin. Consequently, exosomes retained intracellularly in the form of ILVs are subsequently degraded. Arnold M L et al. discovered (Arnold et al., 2023) that in neurons, intermediate filament proteins IFD-1 and IFD-2 colocalize with aggresome-like structures. They promote efficient neuronal exosome generation through a cell-autonomous mechanism. This effect depends on microtubule integrity and dynein function, suggesting that intermediate filaments may indirectly regulate exosome release by participating in aggresome formation and transport. Additionally, vimentin can stabilize the microtubule network through direct interaction with microtubules, inhibiting microtubule depolymerization and promoting their dynamic equilibrium. This provides structural support for the directional transport of exosomes along microtubules. Therefore, there is a natural structural adaptability in the interaction between the cytoskeleton and exosomes: First, the polar tubular structure of microtubules provides an intracellular track for the long-distance directional transport of MVBs that can achieve precise spatiotemporal regulation, and its dynamic instability can directly regulate the transport efficiency and fate of MVBs. Second, the dynamic polymerization-depolymerization characteristics of microfilaments can generate local contractile force, providing a power source for the anchoring, fusion and final exocytosis of MVBs with the plasma membrane, and can control the site and efficiency of exosome release by remodeling the cortical microfilament layer. Third, intermediate filaments form physical cross-links with microtubules and microfilaments to stabilize the spatial structure of intracellular transport tracks, and maintain the intracellular homeostasis during MVB maturation by anchoring organelles, providing a stable intracellular microenvironment for exosome biogenesis.

### 3.3 Influence of exosomes on cytoskeleton

Exosomes regulate cytoskeletal reorganization in recipient cells by delivering various bioactive molecules. This process involves multiple signaling pathways and dynamic changes in cytoskeletal components. Regarding microfilaments, Transforming Growth Factor-β (TGF-β) carried by exosomes binds to TGF-β receptors on the recipient cell surface. This activates the Smad signaling pathway, upregulating the expression of cytoskeleton-related genes like Alpha Smooth Muscle Actin (α-SMA). Consequently, it promotes the formation of microfilament stress fibers (Rodrigues-Junior et al., 2022; Jia et al., 2024). Wu S et al. found (Wu et al., 2022) that the lncRNA UCA1 in exosomes can enter recipient cells and form a ternary complex with Ubiquitin Specific Peptidase 14 (USP14) and the actin-binding protein profilin 1 (PFN1). This complex inhibits the ubiquitination-mediated degradation of PFN1, thereby continuously activating the RAS Homolog Gene Family, Member A (RhoA)/Rho-associated coiled-coil containing protein kinase (ROCK) signaling pathway, promoting the assembly of microfilament stress fibers, and ultimately inducing endothelial cell injury. Jia R et al. discovered in a liver fibrosis model (Jia et al., 2024) that exosomal membrane proteins can reduce α-SMA expression by inhibiting the TGF-β/Smad pathway, thereby reversing microfilament reorganization in fibrotic cells. Regarding microtubules, studies have confirmed (Weiner et al., 2020) that Wnt signaling proteins colocalize with the early endosome marker Rab5. At dendritic branch points, they regulate the localization of γ-tubulin, thereby promoting microtubule nucleation and dynamics. Yan et al. (2025) found that mesenchymal stem cell-derived exosomes can act on the Wnt signaling pathway. Through the Wingless/Integrated (Wnt)/β-catenin pathway, they inhibit the TGF-β/α-SMA fibrotic pathway, influence cytoskeletal reorganization, and promote multi-organ regeneration. Dong B et al.'s research showed (Dong et al., 2025) that cytoskeletal disruption (e.g., depolymerization of F-actin, α-tubulin, β-tubulin) triggers cell death. However, the ESCRT-III complex associated with exosome secretion (especially the CHMP3/CHMP5 subunits) can maintain microfilament cytoskeleton integrity by repairing plasma membrane damage. This prevents cell disintegration due to excessive vacuolization, which indirectly confirms the impact and importance of exosomes on the cytoskeleton. Regarding intermediate filaments, vimentin carried by exosomes plays a key role in regulating intermediate filament homeostasis. Exosomes derived from adipose progenitor cells can protect fibroblasts from osmotic stress, inhibit apoptosis, and promote the reorganization of the intermediate filament network during wound healing by delivering vimentin (Parvanian et al., 2021). Zhou S et al. found (Zhou et al., 2020) that when astrocyte-derived exosomes were directly applied to the ventricles, brain endothelial cell-derived exosomes increased the number of BrdU/nestin-positive cells in specific brain regions of rats. Nestin, a protein specific to neuroepithelial stem cells, belongs to the intermediate filament family of the cytoskeleton. This indicates that exosomes can effectively act on the cytoskeleton, promoting its reorganization and expression. Exosomes can achieve precise reverse regulation of the three major cytoskeletal components (microtubules, microfilaments, and intermediate filaments) of recipient cells through the carried bioactive molecules such as proteins and nucleic acids. They induce cytoskeletal rearrangement through multiple signaling pathways such as TGF-β/Smad, RhoA/ROCK, and Wnt/β-catenin, thereby regulating the core biological functions of recipient cells such as morphology, migration, and proliferation. This reverse regulation process constitutes the core downstream link of the two-way interaction between them, and is also the key molecular basis for exosomes to realize intercellular communication.

#### 3.3.1 Synergistic regulatory network of exosomes on the cytoskeleton

Microfilaments, microtubules and intermediate filaments do not function independently in the cytoskeleton system, but form a highly integrated and dynamically coordinated three-dimensional network structure through two core dimensions: physical cross-linking and signal cascade coupling (Pradeau-Phélut and Etienne-Manneville, S., 2024; Conboy et al., 2024; Ndiaye et al., 2022). As a key medium of intercellular communication, exosomes do not only independently regulate a single cytoskeletal component, but realize the overall regulation of the cytoskeleton system of recipient cells by targeting the core nodes of the synergistic network (Dai et al., 2020; Chen et al., 2016; Sung et al., 2021); this synergistic regulatory mechanism is the core molecular basis for exosomes to drive complex cell biological behaviors such as cell migration, morphological remodeling, and mechanical signal transduction (Conboy, J. P. et al., 2024; Parvanian, S. et al., 2025; Liu et al., 2022). Rho family small GTPases (with RhoA, Rac1, and Cdc42 as the core) are the core signal cascade hub for exosomes to synergistically regulate the three major cytoskeletal components. As the “master switch” of cytoskeletal dynamic changes, Rho family small GTPases can simultaneously act on microfilaments, microtubules and intermediate filaments through downstream effector molecules, realizing the spatiotemporal synchronization of cytoskeletal rearrangement (Hervé and Bourmeyster, 2015; Yan et al., 2020; McAtee et al., 2025; Mosaddeghzadeh and Ahmadian, 2021; Kajimoto et al., 2018). Exosomes derived from trophoblast cells can deliver lncRNA UCA1 to endothelial cells, and form a ternary complex with ubiquitin-specific peptidase 14 (USP14) and actin-binding protein profilin 1 (PFN1), inhibiting the ubiquitination degradation of PFN1, and continuously activating the RhoA/ROCK signaling pathway (Sung et al., 2021); this RhoA/ROCK signaling pathway can achieve synergistic remodeling through targeted regulation of the three major cytoskeletal components of microfilaments, microtubules and intermediate filaments, thereby inducing endothelial cell injury and dysfunction. The regulation of microfilaments is mainly reflected in promoting the assembly of microfilament stress fibers and mediating microfilament rearrangement, and its regulatory effect is completed through two core pathways: Rho-associated coiled-coil containing protein kinase/Myosin Phosphatase Targeting Subunit 1/Myosin Light Chain (ROCK/MYPT1/MLC) and Rho-associated coiled-coil containing protein kinase/LIM Kinase/Cofilin (ROCK/LIMK/cofilin) (Amano et al., 1996; Maekawa et al., 1999; Wilson et al., 2005; Ohashi et al., 2000); the regulation of microtubules is achieved by phosphorylating MAPs, which can directly regulate the dynamic instability of microtubules. Among them, CRMP2, MAP2, and Tau are its key phosphorylation targets, and MAP2 and Tau have been confirmed to be direct substrates of ROCK (Amano et al., 2003; Arimura et al., 2005); for the regulation of intermediate filaments, this pathway can specifically mediate the phosphorylation modification and network rearrangement of vimentin, and the core target of its phosphorylation regulation is the Ser71 site of vimentin, while the Ser56 site is mainly phosphorylated by PAK1 (Goto et al., 1998; Wang et al., 2007). In addition to the above regulatory effects mediated by RhoA, Rac1 and Cdc42 of the same family are also key molecules for exosomes to regulate the cytoskeletal synergistic network, which mediate cytoskeletal remodeling and cell movement processes through specific signal axes respectively. Bioactive molecules carried by exosomes can activate Rac1, and then activate its downstream effector molecules PAK and Arp2/3 complex, driving the formation of lamellipodia at the leading edge of the cell and the assembly of branched microfilament network, and regulating the forward extension and dynamic stability of microtubules, which is one of the core regulatory factors for exosomes to induce directional migration of recipient cells (Gopal et al., 2016; Fujioka et al., 2025; Ma et al., 2023; Garcin and Straube., 2019). At the same time, exosomes can also drive the formation of filopodia and the establishment of cell polarity by targeting the activity of Cdc42, and regulate the network assembly of intermediate filaments and the nucleation process of microtubules through downstream effector molecules, playing an irreplaceable role in the spatial orientation of the cytoskeleton, invasion and morphological remodeling of recipient cells (Liu, Y. et al., 2021; McAtee, C. et al., 2026; Fukata, M. et al., 2002; Leduc, C. and Etienne-Manneville, S., 2017). If the signal cascade mediated by Rho family small GTPases is the “command center” for the synergistic rearrangement of the cytoskeleton, then cytoskeletal cross-linking proteins are the “structural bridge” to realize the physical coupling and mechanical transmission of the three major cytoskeletal components, which together constitute the core system for exosomes to regulate the cytoskeletal synergistic network. Exosomes, as key functional carriers for intercellular information transmission, play an important role in cell physiological and pathological processes, and cytoskeletal cross-linking proteins are the core structural basis for realizing the physical connection and functional synergy between different cytoskeletal components (Leung et al., 2002; Steinböck and Wiche, 1999; Suozzi et al., 2012). Their synergistic effect jointly regulates a variety of physiological and pathological activities of cells. Among them, the plakin family (with plectin as the core representative) is the most important cytoskeletal cross-linking molecule in eukaryotic cells. It can simultaneously bind to the three major cytoskeletal components of actin microfilaments, microtubules and intermediate filaments through its multi-domain characteristics, bridging them into a network structure with complete mechanical conduction characteristics, thereby completing the spatial integration and functional synergy of the cytoskeleton system (Leung et al., 2002; Steinböck and Wiche, G., 1999; Suozzi et al., 2012). Existing *in vivo* and *in vitro* experimental studies have confirmed that exosomes can indirectly participate in the dynamic regulation of the cytoskeleton by targeting the expression level of core members of the plakin family (McAtee et al., 2026; Salih et al., 2016; Lin B et al., 2021), and this regulatory process is often achieved by mediating the expression of microRNA, among which miR-124-3p is one of the important regulatory mediators. Specifically, exosomes can regulate the expression of miR-124-3p, which can block the transformation of epithelial cells into pro-fibrotic mesenchymal cells, thereby slowing down and reducing renal fibrosis (Chen, G. et al., 2024; Xia et al., 2023; Zhang et al., 2024); at the same time, it has also been found in tumor research that miR-124-3p can directly target plectin, the core member of the plakin family, thereby inducing cytoskeletal remodeling (Deng et al., 2021; Nolan et al., 2020). In addition to the plakin family that can bridge the three major cytoskeletal components at the same time, the ezrin/radixin/moesin (ERM) protein family, which mediates cell membrane-cytoskeleton anchoring and mechanical signal transduction, is also a key target for exosomes to regulate the cytoskeletal synergistic network. The ERM protein family is also the core functional molecule and key intervention target of the cytoskeleton synergistic regulatory network. As the core cross-linking molecule connecting the cell membrane and microfilaments, ERM protein mainly mediates the connection between the cell membrane and microfilaments and mechanical signal transduction, and is a key molecule for maintaining cell morphology and membrane structure stability (Kawaguchi et al., 2017). The high expression of ERM protein is associated with the poor prognosis of various tumors (Clucas and Valderrama, 2014). In gliomas, the abnormal expression and activity of ezrin, the core member of the ERM family, is the key mechanism driving the invasion and malignant progression of glioma cells (Wick et al., 2001); exosomes derived from glioma cells can regulate the phosphorylation level of ezrin by delivering circGLIS3 (hsa_circ_0002874), thereby promoting the invasion and progression of glioma (Li et al., 2021), which suggests the potential connection between exosomes and the synergistic regulatory network of the cytoskeleton. In summary, exosomes mainly achieve the overall regulation of the cytoskeleton synergistic network through two core paths: one is the signal cascade regulation centered on Rho family small GTPases, which realizes spatiotemporally coordinated cytoskeletal rearrangement by synchronously targeting the three major components of microfilaments, microtubules and intermediate filaments; the other is the physical coupling regulation centered on cytoskeletal cross-linking proteins, which realizes the structural integration and mechanical conduction synergy of the three major cytoskeleton networks by regulating the expression and activity of molecules such as plectin and ERM family. Therefore, exosomes do not solely regulate a certain component of the cytoskeleton, but take Rho family small GTPases as the signal cascade hub and cytoskeletal cross-linking proteins as the structural bridge to realize the synergistic overall regulation of microtubules, microfilaments and intermediate filaments through the two core paths. This synergistic regulatory network is the core mechanism for exosomes to drive complex biological behaviors of cells, and also fully reveals the whole picture of molecular regulation of the two-way interaction between them, lifting the research on the interaction mechanism between them from single component regulation to the level of network synergistic regulation. The core mechanisms of key proteins and signaling pathways involved in this exosome-cytoskeleton interaction are summarized in Table 1.

**TABLE 1:** Summary of the core mechanisms of key proteins and pathways in the interaction between exosomes and the cytoskeleton.

Category	Name	Core mechanism in the interaction between exosomes and the cytoskeleton
Key regulatory proteins	Vimentin	As the core component of intermediate filaments, it is the key mediator of the interaction between the two: ① In donor cells, it provides structural support for the directional transport of MVBs by anchoring organelles and stabilizing the microtubule network, and directly promotes exosome release; ② it can be carried and delivered to recipient cells by exosomes, regulating the reorganization of the intermediate filament network in recipient cells, and mediating effects such as epithelial-mesenchymal transition and osmotic stress protection
	α-Smooth muscle actin (α-SMA)	The core effector molecule of cytoskeletal rearrangement: exosomes regulate its expression level through the TGF-β/Smad signaling pathway, thereby driving the formation of microfilament stress fibers in recipient cells. It is the core executive protein for exosomes to induce cytoskeletal remodeling and regulate cell contraction and migration functions
	Cortactin	A key actin-binding protein that regulates exosome secretion in donor cells: it promotes the formation of branched microfilament by activating the Arp2/3 complex, stabilizes the docking sites of MVBs on the plasma membrane, and directly enhances the efficiency of exosome release. It is the core node for microfilament cytoskeleton to regulate exosome secretion
	Kinesin/Dynein	Microtubule-dependent molecular motors, which are the core executive molecules for cytoskeleton regulation of exosome secretion in donor cells: they respectively mediate the directional transport of MVBs along microtubules to the cell periphery (plus end) and perinuclear region (minus end). The dynamic balance between the two directly determines the intracellular localization of MVBs and the efficiency of exosome secretion
​	Ubiquitin specific peptidase 14 (USP14)/Profilin 1 (PFN1)	Key molecules for exosomes to regulate cytoskeletal rearrangement in recipient cells: lncRNA UCA1 carried by exosomes can mediate the formation of a ternary complex between them, inhibit the ubiquitination degradation of PFN1, and then activate the RhoA/ROCK signaling pathway, promoting the assembly of microfilament stress fibers and cytoskeletal rearrangement in recipient cells
	Rab GTPases family (Rab27a/b, Rab7, Rab11a, Rab35)	The core molecular switch for cytoskeleton regulation of exosome secretion: Through the GTP/GDP cycle state, it precisely coordinates the whole process of directional transport of MVBs along microtubules, plasma membrane anchoring and fusion. Among them, Rab27b mediates the transport of MVBs along microtubules to the cell periphery, Rab27a regulates the docking of MVBs with the plasma membrane, Rab7 mediates the transport of MVBs to the minus end of microtubules in lysosomes, which directly determines the efficiency of exosome secretion
	Core proteins of ESCRT complex (Alix, TSG101, VPS4)	The core regulatory proteins of exosome biogenesis, which are highly coupled with the dynamic regulation of the cytoskeleton: they mediate endosomal membrane invagination and ILVs formation, and complete MVB maturation. The intracellular transport and fate decision of mature MVBs are highly dependent on the synergistic regulation of microtubule/actin cytoskeleton, which is the upstream core target of cytoskeleton regulation of exosome generation
	Tetraspanins (CD9, CD63, CD81)	Hallmark proteins of exosomes, participating in ESCRT-independent exosome biogenesis: they regulate endosomal membrane curvature and ILVs formation, and at the same time regulate membrane fluidity by sorting cholesterol, affecting the transport efficiency of MVBs along the cytoskeleton and the fusion ability of exosomes with target cell membranes
	Ezrin/Radixin/Moesin (ERM family)	The core structural bridge for exosomes to regulate the cytoskeletal synergistic network: it connects the cell membrane and microfilaments, mediates mechanical signal transduction. Exosomes derived from tumor cells can regulate the phosphorylation level of its core member ezrin through circRNA, thereby driving cytoskeletal rearrangement and tumor invasion
​	Plectin	The core cross-linking molecule of the three major components of the cytoskeleton: it can simultaneously bind to microtubules, microfilaments and intermediate filaments to realize the structural integration of the cytoskeleton network. Exosomes can target its expression by regulating miR-124-3p, thereby inducing the overall cytoskeleton network remodeling in recipient cells
Key signaling Pathways/Regulatory pathways	TGF-β/Smad signaling pathway	The core pathway for exosomes to regulate microfilament cytoskeleton rearrangement in recipient cells: After TGF-β carried by exosomes binds to receptors on the surface of recipient cells, it activates Smad molecules, upregulates the transcription of cytoskeleton-related genes such as α-SMA, and induces the formation of microfilament stress fibers. It can also inhibit this pathway through exosomal membrane proteins to reverse the abnormal cytoskeletal remodeling related to fibrosis
	RhoA/ROCK signaling pathway	The core signal hub for exosomes to synergistically regulate the three major cytoskeletal components of recipient cells: Activated by the regulation of molecules such as lncRNA and miRNA carried by exosomes, it can synchronously mediate the assembly of actin stress fibers, the regulation of microtubule dynamic instability, and the phosphorylation and network rearrangement of vimentin. It is the core pathway for exosomes to induce overall cytoskeletal remodeling and regulate cell migration
	Wnt/β-catenin signaling pathway	The key pathway for exosomes to regulate the homeostasis of microtubule cytoskeleton in recipient cells: exosomes can regulate the localization of γ-tubulin through this signaling pathway, promote microtubule nucleation and dynamic changes. At the same time, it can inhibit the TGF-β/α-SMA fibrosis pathway through this signaling pathway, maintain the cytoskeleton homeostasis of recipient cells, and mediate tissue regeneration and repair
​	Microtubule-mediated directional transport pathway of MVBs	The core pathway for cytoskeleton to regulate exosome secretion: Taking microtubules as the “transport track”, relying on the synergistic effect of kinesin/dynein and Rab GTPases, it realizes the long-distance intracellular directional transport of MVBs, which is the key rate-limiting step of exosome secretion. Microtubule depolymerizing agents (such as nocodazole) can significantly reduce exosome release by inhibiting this pathway
	Microfilament-mediated MVB-plasma membrane fusion pathway	The core pathway for cytoskeleton to regulate exosome secretion: The contractile force generated by microfilament rearrangement provides power for the fusion of MVBs with the plasma membrane. At the same time, it can promote the anchoring, fusion of MVBs with the plasma membrane and exosome exocytosis release by reducing the density of cortical actin at the MVBs anchoring sites
	ESCRT-dependent/Independent Exosome biogenesis pathway	The core pathway of exosome generation, which are highly coupled with the dynamic regulation of the cytoskeleton: The ESCRT-dependent pathway, the N-SMase2/ceramide-mediated ESCRT-independent pathway, and the tetraspanin-mediated pathway jointly complete the biosynthesis of ILVs and MVBs. The fate decision and intracellular transport of mature MVBs are highly dependent on the synergistic regulation of the cytoskeleton system

## 4 Conclusion

The dynamic two-way interaction between exosomes and the cytoskeleton is the core mechanism that runs through the whole process of intercellular communication and regulates cellular physiological homeostasis and pathological processes (van Niel et al., 2018; Ostrowski et al., 2010; Xu et al., 2023). The two-way regulatory network formed by them is precise and orderly, which is specifically manifested in the following two aspects: On the one hand, the dynamic network composed of the three major components of the cytoskeleton is the core basis for regulating various biological behaviors of exosomes. Among them, microtubules provide polar track support for the long-distance intracellular directional transport of MVBs encapsulating ILVs (Xu et al., 2023; Barlan and Gelfand, 2017; Knudsen et al., 2023), and the dynamic rearrangement of microfilaments provides the core contractile force for the anchoring and fusion of MVBs with the plasma membrane (Sinha et al., 2016; Calvo and Izquierdo, M., 2021); while intermediate filaments (such as vimentin) indirectly regulate the secretion efficiency of exosomes by stabilizing the cytoskeleton network and anchoring organelles (Wu et al., 2021; Arnold et al., 2023). On the other hand, exosomes can induce the overall rearrangement of the cytoskeleton in recipient cells by delivering bioactive molecules such as proteins and nucleic acids through key signaling pathways such as the TGF-β/Smad signaling pathway, the RhoA/ROCK signaling pathway, and the Wnt/β-catenin signaling pathway, thereby regulating the core biological functions of target cells such as morphology maintenance, migration and proliferation (Rodrigues-Junior et al., 2022; Jia et al., 2024; Wu et al., 2022; Yan et al., 2025). This two-way interaction process relies on the synergistic regulation of core molecules such as Rab GTPases family and SNARE complex, forming a precise “regulation-feedback” dynamic balance system (Ostrowski, et al., 2010; Xu et al., 2023; Fader et al., 2009). Its physiological homeostasis maintains the normal life activities of the body, while the imbalance of regulation is closely related to the occurrence and development of various diseases such as tumors, neurodegenerative diseases, and atherosclerosis (Sung and Weaver, 2017; Malm et al., 2016; Peng et al., 2022).

At present, the research on the interaction between exosomes and the cytoskeleton is gradually expanding from the basic molecular mechanism to the field of clinical translation, but there are still many key scientific problems to be solved in this field. First of all, existing studies mostly focus on the dominant functions of microtubules and microfilaments in the interaction, and the exploration of the regulatory role of the intermediate filament family is still weak. As core components of the cytoskeleton, members of the intermediate filament family (such as desmin specifically expressed in muscle cells, neurofilament proteins in neuronal axons) have unique mechanical sensing and organelle anchoring functions. Existing studies have only confirmed that vimentin can participate in exosome secretion and cytoskeleton regulation in recipient cells (Pogoda et al., 2022; Eriksson et al., 2009; Wu et al., 2021; Parvanian et al., 2021), but the functions of other members of the family, the cross-regulatory network with microtubules/microfilaments, and the influence of phosphorylation modification on the interaction process lack systematic experimental evidence and in-depth mechanistic analysis. Secondly, the targeting specificity mechanism of the reverse regulation of exosomes on the cytoskeleton has not yet been clarified. The cell type-dependent heterogeneity of exosome contents has been widely confirmed (Zhang et al., 2020; Li X. et al., 2023), but how the cargo molecules of exosomes from different sources precisely activate the cytoskeleton regulatory signaling pathways in recipient cells, and the molecular basis of their sequence specificity and targeted binding remain to be analyzed (Zhang et al., 2020; Li et al., 2023; Lin H et al., 2021); the structural basis of exosome lipid components regulating cytoskeleton rearrangement through a receptor-independent manner, and the kinetic process of their synergistic action with protein cargo still need to be further explored combined with multi-omics and structural biology techniques (Skotland et al., 2019; Kalluri and LeBleu, 2020; Palmulli et al., 2024). In addition, most existing studies are carried out based on *in vitro* cell lines, and there is still a lack of sufficient *in vivo* experimental evidence to support the real regulatory rules and cell type-specific effects of the interaction between the two in the *in vivo* physiological and pathological microenvironment.

In response to the above problems, future research needs to focus on three core directions: First, with the combined use of single-cell sequencing, spatial proteomics and ultra-high resolution imaging technology, construct a full regulatory network map of exosome cargo-cytoskeleton signaling pathway, focus on exploring new regulatory factors related to the intermediate filament family, and analyze the spatiotemporal coordination rules of the three major cytoskeleton components in the interaction process (Pradeau-Phélut and Etienne-Manneville, 2024; Ndiaye et al., 2022). Second, develop a bispecific intervention strategy targeting the cytoskeleton-exosome interaction, for example, design targeted molecules that can simultaneously regulate cytoskeleton dynamics and exosome secretion, or use exosomes to deliver cytoskeleton regulatory drugs to achieve precise treatment of diseases, and promote the translation of basic research into clinical applications (Deng et al., 2021; Li et al., 2021). Third, combine organoid models and gene-edited model organisms to verify the core mechanism of the interaction between the two in the *in vivo* physiological and pathological microenvironment, clarify its causal relationship in the occurrence and development of diseases, and provide new targets and strategies for disease diagnosis and treatment.

In summary, the research on the interaction between exosomes and the cytoskeleton is at the intersection of basic theoretical breakthroughs and clinical transformation applications. In-depth analysis of the molecular mechanism of the two-way interaction between them can not only improve the basic biological theory of intercellular communication, but also provide innovative ideas for the precise diagnosis and treatment of various refractory diseases such as tumors, fibrosis, and neurodegenerative diseases, which has important scientific value and clinical transformation prospects.
